# Translating the hemodynamic response: why focused interdisciplinary integration should matter for the future of functional neuroimaging

**DOI:** 10.7717/peerj.6621

**Published:** 2019-03-25

**Authors:** Sigita Cinciute

**Affiliations:** Institute of Biosciences, Life Sciences Center, Vilnius University, Vilnius, Lithuania

**Keywords:** Cerebrovascular regulation, Healthcare, Hemodynamic response, Neuroscience, Brain, Computational modelling, Functional near-infrared spectroscopy, Neurovascular coupling, Functional magnetic resonance imaging

## Abstract

The amount of information acquired with functional neuroimaging techniques, particularly fNIRS and fMRI, is rapidly growing and has enormous potential for studying human brain functioning. Therefore, many scientists focus on solving computational neuroimaging and Big Data issues to advance the discipline. However, the main obstacle—the accurate translation of the hemodynamic response (HR) by the investigation of a physiological phenomenon called neurovascular coupling—is still not fully overcome and, more importantly, often overlooked in this context. This article provides a brief and critical overview of significant findings from cellular biology and in vivo brain physiology with a focus on advancing existing HR modelling paradigms. A brief historical timeline of these disciplines of neuroscience is presented for readers to grasp the concept better, and some possible solutions for further scientific discussion are provided.

## Introduction

Modern functional neuroimaging methods cover broad spatial and temporal scales (Pouratian et al., 2003) and facilitate the important exploration of the functional organisation of the human brain in health and disease (Liu et al., 2015). Numerous statistical or methodological challenges are addressed with this complexity. However, some threats arise from fundamental conceptual challenges that remain widely underappreciated within the clinical and neuroimaging communities (Poldrack & Yarkoni, 2016). Current trends in neuroimaging and computational neuroscience promote the advanced mathematical modelling of human brain function based on neuroimaging, and the implications associated with the use of Big Data (Hansen et al., 2014) in scientific research and healthcare innovations. These multidisciplinary interactions between different branches of science are vital for overall scientific progress. However, some main conceptual challenges may remain shadowed by massive trends and become a barrier to progress. For example, the ability to assess neural activity in a non-invasive way by measuring the brain’s circulation of blood has revolutionised neuroscience. As a result, we are witnesses to enormous growth in the field of human brain research (Raichle, 2009; Toga, 2015). Each of the functional neuroimaging techniques used today, such as functional near-infrared spectroscopy (fNIRS), functional magnetic resonance imaging (fMRI), positron emission tomography (PET) or single photon emission computed tomography (SPECT) explore different metabolic or particular physiological events, but all are based on the physiological principles of neurovascular coupling (NVC). NVC is the process by which active brain regions induce a local increase in blood flow to match their energy demands via the dilation of capillaries and arterioles through various cellular signalling paths (Mishra et al., 2016). Capillary dilation generates a significant portion of the blood flow increase, evoked by neuronal activity (Hall et al., 2014), and is expected to contribute substantially to the observed hemodynamic response (HR) (Lindauer et al., 2010). Nevertheless, our understanding of NVC in humans despite its importance is still incomplete due to a lack of appropriate and consistent analysis strategies and stimulation paradigms (Hillman, 2014; Phillips et al., 2016). Despite their technological differences, widely used functional neuroimaging techniques, such as fMRI and less known but prominent fNIRS, are mainly based on the common underlying phenomenon of NVC (Hillman, 2014; Huneau, Benali & Chabriat, 2015; Iadecola, 2017; Phillips et al., 2016). It makes some results of current experimental methods ambiguous compared to the more in-depth fundamental perspective (Lindauer et al., 2010; Phillips et al., 2016; Sotero & Trujillo-Barreto, 2007).

The scientific knowledge is always limited to some extent and arguably with each new finding. However, the real concerns occur summarising that at the moment, a big part of scientific and clinical research production is based on sophisticated mathematical manipulations of neuroimaging data which was derived from the observations of NVC, referred to as a HR. A generalisation like this emphasises the need of tighter and focused interdisciplinary integration within particular neuroscience fields to improve the translation of physiological signal into neuroimaging data, which later is processed with sophisticated mathematical and statistical methods. The goal of this article was to fill the gap between the critical and brief overviews of one of the under-appreciated neuroimaging challenge: accurate translation of the HR to scientific and clinical findings. The next objective was to provide the scientific reader with a summary of key aspects and analyse why this problem is relevant to those that are interested in or directly involved in human cognition and behaviour neuroscience or clinical research. To serve this purpose, this article conceptualises the current knowledge of NVC from several perspectives, mainly cellular and molecular neuroscience (CMN), and functional neuroimaging (particularly fNIRS and fMRI as they are closely related).

## Survey Methodology

The review was designed with a focus on the existing scientific paradigm in human brain functional neuroimaging research, mainly addressing the absence of necessary interactions between different multidisciplinary branches of neuroscience such as cellular biology, human brain physiology and computational modelling (CM). The articles that were reviewed in this paper were identified in databases (e.g. Google Scholar, PubMed, ScienceDirect) and subject-specific professional journals and websites (e.g. PLOS, PeerJ, Frontiers, Journal of Cerebral Blood Flow & Metabolism). The literature review was assured to be unbiased and comprehensive by narrowing down the exploration by searching for original research articles and reviews that discuss (i) cerebrovascular regulation; functional hyperaemia; NVC; astroglial network; the origin of the HR signal in fNIRS; the origin of the blood-oxygen-level-dependent (BOLD) signal in fMRI; or (ii) the biophysical model of fNIRS and fMRI signals; the CM methods used in fNIRS and fMRI; and (iii) studies that compare both methods or combine them in humans. The author also searched for articles in cellular and molecular biology, animal studies, subsequently regarding studies in health and disease. However, it was done only in combination with the previously mentioned search criteria to identify relevant publications. The other inclusion criteria for selected articles required that articles would be directly related to the topic and would not exhaustively cover unrelated material such as other neuroscience methods if the results were not directly comparable with functional neuroimaging.

## What is a Hemodynamic Response?

The human brain represents only 2% of the total body mass. About 25% of the oxygen and from 20% to 70% of the glucose consumed by the human body is dedicated to cerebral functions (Herculano-Houzel, 2011). The maintenance and restoration of the ion gradients dissipated by signalling processes such as post-synaptic and action potentials, as well as the uptake and recycling of neurotransmitters are the primary processes contributing to the high brain’s energy needs (Attwell et al., 2010). The brain blood circulation system actively regulates the constant demand and supply. However, active brain regions are often provided more than they require. One of the researchers poetically illustrated it as ‘watering the garden for the sake of a single thirsty flower’ (Malonek & Grinvald, 1996). This overcompensation or functional hyperaemia is a fundamental phenomenon in normal brain function. It was first confirmed by (Roy & Sherrington, 1890) and defines the dilation of arterioles and capillaries of a brain region in response to a local episode of high neuronal activity. Functional hyperaemia is a generalised term for the outcome of a complex cerebrovascular regulation mechanism which will be briefly discussed in this section. At this point, the term HR is associated with the quantitative measures of functional hyperaemia using fNIRS and fMRI. In fMRI, it is better known as the HR function (HRF) to imply its mathematical properties. Further, in the text, only the HR term will be used, as it describes an observation of a physiological NVC event common for both techniques.

**Figure 1:**
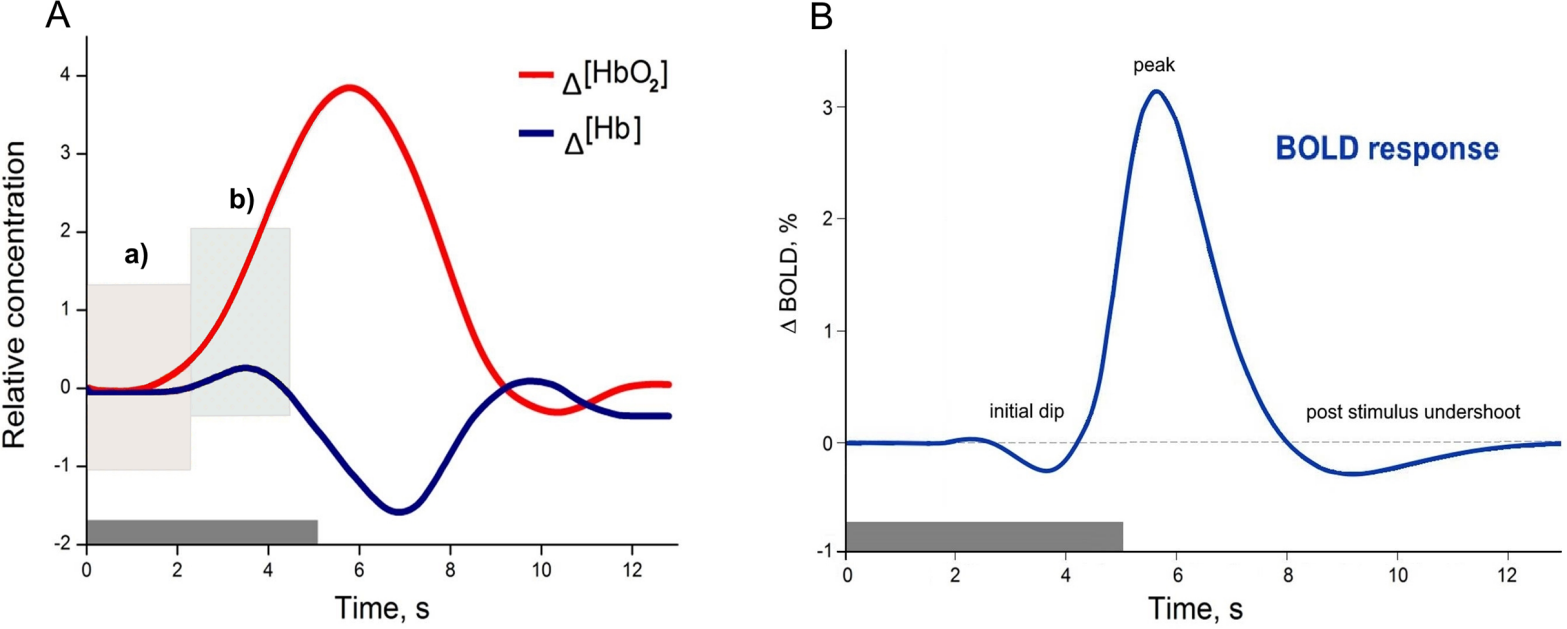
Examples of canonical hemodynamic response (A), and hemodynamic response function (B). A neural activity from 0 to 5 s (grey bar) causes neurometabolic and later neurovascular coupling, which can be seen as a delay of response (around 2 s). Box in hemodynamic response (A) indicates (a) small inflow of Δ(HbO_2_), when the total blood volume is still relatively unchanged (due to increased cerebral blood flow), and later (b) Δ(HbO_2_) increases rapidly due to functional hyperemia caused by vasodilatation. The small increase of Δ(Hb) occurs due to insufficient washout when the cellular oxygen demand exceeds current supply in a tissue. The canonical example of a hemodynamic response is based on measuring the composition of cerebral blood volume via chromophore concentration changes (oxy-Hb and deoxy-Hb). fNIRS studies can directly measure both oxy-Hb and deoxy-Hb), (Venclove, Daktariunas & Ruksenas, 2015). In contrast, the canonical hemodynamic response function (HRF) from the Blood Oxygenation Level Dependent (BOLD) method represents the magnetic field change in response to the Δ(Hb) curve (B) and is relative to the baseline.

Functional magnetic resonance imaging and fNIRS have different capacities to explore human brain functions. As was introduced, both methods, despite their data acquisition differences, are based on a common underlying phenomenon termed NVC. However, fMRI is more common in general, due to its broad application possibilities and historical background, especially in clinical practice (Glover, 2011), while fNIRS was primarily used for the bedside monitoring of infants, and other fields where fMRI were not applicable. Thus only quite recently with technical progress fNIRS became an equivalent method for investigating human cognitive brain functions (Boas et al., 2014). Despite the common underlying phenomenon (Hillman, 2014; Huneau, Benali & Chabriat, 2015; Iadecola, 2017; Phillips et al., 2016) both methods have their own strengths and limitations associated with biophysical and physiological signal origins (Kim & Ogawa, 2012; Scarapicchia et al., 2017). A comparison of how the same physiological process of NVC originates as a HR measured using fNIRS (A) and HR function measured using fMRI (B) can be found in Fig. 1. The example of HR (A), is based on measuring the composition of the total cerebral blood volume in the particular brain area. These measures can be directly done in vivo, and both oxygenated haemoglobin Δ(HbO_2_) and deoxygenated haemoglobin Δ(Hb) concentration changes are observed simultaneously. In contrast, the BOLD signal in fMRI is based on the paramagnetic deoxyhemoglobin decrease in T2* contrast, relative to the situation with diamagnetic oxyhemoglobin (Bandettini & Wong, 1995; Chavhan et al., 2009). Thus, the Δ(Hb) curve in Fig. 1A could be seen as a BOLD signal representation, even though it is not straightforward due to other physiological contributors captured in the BOLD response (such as regional cerebral blood flow and volume), (Arthurs & Boniface, 2002; Kim & Ogawa, 2012).

With technological progress, the number of combined functional fMRI-fNIRS studies has begun to increase slowly. According to the PubMed database search for the terms ‘fMRI’ and ‘fNIRS’ in the title/abstract field and restricted to the results of the articles and review papers published between January 1, 1977, and June 6, 2018, a total of 752 documents with human participants were identified. After additional restriction to search for these terms only in the title field, a total of 11 documents remained (Anwar et al., 2013; Cui et al., 2011; Frederick, Nickerson & Tong, 2012; Gagnon et al., 2012; Maggioni et al., 2013; Okamoto et al., 2004; Sasai et al., 2012; Sato et al., 2013; Strangman et al., 2002; Tong & Frederick, 2010; Toyoda et al., 2008). A few of them were comparisons, which have been conducted for a variety of cognitive tasks to illustrate similarities and differences between fNIRS and fMRI capabilities (Cui et al., 2011; Steinbrink et al., 2006). Overall, the described PubMed search serves as an illustrative example for two points: (a) number of multimodal, cross-validation, or comparison studies within even closely related techniques such as fMRI, and fNIRS is still insufficient; and (b) that integration of more distinct branches of neuroscience might be even more daring, but as will be discussed in the further section, might also add compelling value to the field of functional human brain research.

### Hemodynamic response and classical approach in neuroimaging

The fundamentals of coupling between brain electrical activity, metabolism, and the observed HR are incredibly complex. Classical functional neuroimaging approaches for simplicity assume that the vascular response induced by neural activity is a nearly linear function of blood volume increase. This approach is convenient to model and reconstruct the possible neural activity from the HR but is not entirely accurate. Nonlinearities are believed to arise from both nonlinearities in the vascular response and neuronal activity, as several studies have demonstrated (Birn et al., 2013; Birn & Bandettini, 2005; Hillman, 2014; Wager et al., 2005). Many more studies may be discussed depending on the reader’s point of view, but the two following studies were chosen subjectively to provide a short illustrative introduction to this topic for non-experts. Wager et al. (2005) an empirical study with fMRI, where they attempt to characterise nonlinear effects in visual and motor cortex in 12 human participants, presents finding that these nonlinear effects are relatively consistent throughout the tested brain areas. Additionally, a more recent and exciting multimodal study by Fabiani et al. (2014) published in 2014 is recommended. In this multimodal study, 19 young and 44 older healthy adults were examined to address physical fitness and age effects on NVC in the primary visual cortex and show the quadratic relationship between neural activity and blood flow. The overall results indicate that nonlinearity in NVC has more than one aspect to be considered, and the classical neuroimaging approach is not sufficient to explain it.

What is the classical explanation of the origin of the HR? Over a decade, the understanding of NVC initiation and its overall role for HR formation dramatically changed (Attwell et al., 2010). From a CMN perspective, for a long time, researchers favoured the idea that blood flow is locally controlled by a passive negative-feedback system, where neural activity leads to a local substrate demand. It was thought that the metabolic signal inducing HR could be a lack of O_2_ or glucose, or the local rise of CO_2_, ADP or lactate. However, this negative-feedback hypothesis failed to adequately explain the experimental findings of NVC in animal models (Attwell et al., 2010; Walsh, 2016). Manipulations of O_2_ and glucose did not regulate blood flow as expected, and CO_2_, ADP together with lactate showed only partial effects (Attwell et al., 2010). More novel, feed-forwards neurotransmitter-mediated mechanisms suggest active control of the vascular energy supply in the brain (Attwell et al., 2010). In this process, the neurones either signal or activate astrocytes to release vasoactive messengers onto vessels. According to this hypothesis, astrocytes are anatomical intermediaries between neurones and blood vessels (Attwell et al., 2010; Petzold & Murthy, 2011). However, is it all about astrocytes or other glial cells involved as well? Recent studies have begun to challenge the astrocytes role as the main drivers of NVC due to inconsistencies between spatiotemporal properties of vasodilatation, and the structure-functional properties and distribution of astrocytes in the cortical volume (Iadecola, 2017; McCaslin et al., 2011). In the following sub-section, the three examples of recent in vivo experiments will be discussed, to better illustrate the importance of non-neuronal HR origin.

### What induces a hemodynamic response and why does it matter?

From a historical perspective 100 and 50 years ago the neuroglial was thought to be only a connective material in the brain and was given an entirely passive supportive role (Kettenmann & Verkhratsky, 2008). Since then, a substantial amount of research has been published supporting the idea that the previous ‘neuron-centric’ perspective of neuroscience is not accurate. Today, it is evident that glial cells are integral to the development and maintenance of the healthy central nervous system and play a vital role in the pathogenesis of many brain disorders (Liddelow & Barres, 2017).

The particular scientific focus was first given on astrocytes, as they are the most abundant population of glia in the mammalian brain (Azevedo et al., 2009; Liddelow & Barres, 2017; Von Bartheld, Bahney & Herculano-Houzel, 2016). It was proved that astrocytes are not only responsible for physical brain structuring but also are (a) critical homeostatic cellular elements that are capable of gluconeogenesis, provide neurones with lactate, and control over glucose levels (Bélanger, Allaman & Magistretti, 2011; Brooks, 2009; Magistretti, 2006); (b) form a tripartite synapse with neurones and modulate synaptic activity via ion and neurotransmitters concentrations in the extracellular space (Allen & Eroglu, 2017; Haydon & Carmignoto, 2006; Krencik, Van Asperen & Ullian, 2017; Perea & Araque, 2005; Perea, Navarrete & Araque, 2009); (c) they are responsible for some immune activity, promote neuronal survival and enable re-myelination within the brain (Ayaz et al., 2008; Liddelow & Barres, 2017; Von Bernhardi, 2016); and (d) act as direct and indirect modulators of cerebrovascular tone (Ayata & Lauritzen, 2015; Bazargani & Attwell, 2016; Filosa et al., 2016; Gratton, Chiarelli & Fabiani, 2017; Iadecola, 2017; Mishra, 2017). Moreover, they form an equivalent to neurones astroglial network (Attwell et al., 2010; Giaume et al., 2010; Scemes & Spray, 2003).

After all, one may ask how these new cellular findings translate into functional neuroimaging. For example, the study of in vivo animal models (cat and rat) for a single-vessel hemodynamic demonstrated that pial surface arteries in the cat’s visual cortex (as well as neurones) show orientation responsiveness (in contrast to rats, where orientation maps are not shown in general), meaning that propagation of vascular dilation between neighbouring columns in the brain needs to be accounted for when decoding hemodynamic signals (O’Herron et al., 2016).

Another in vivo study of animal models (rat and mice) show that when the sensory input increases, blood flow capillaries dilate before arterioles and are estimated to produce 84% of the blood flow increase (Hall et al., 2014). Previously, it was thought that capillaries usually do not significantly contribute. Moreover, the study identifies pericytes as significant regulators of cerebral blood flow as they are the first vascular elements to dilate during neuronal activity, and, in turn, initiate hyperaemia. It also unexpectedly showed that vasodilators released from active neurones, interneurones and astrocytes (Hamel, 2006; Miller & Halpern, 2014) are not the only essential players in functional imaging. In fact, the role of pericytes in CNS is as diverse as it was previously described with astrocytes: pericytes integrate, coordinate and process signals from their neighbouring cells to generate diverse functional responses that are critical for CNS functions in health and disease, including (a) regulation of the blood-brain barrier (BBB) permeability; (b) angiogenesis; (c) clearance of toxic metabolites; (d) neuroinflammation and stem cell activity; and finally (e) initiating capillary HRs (Hall et al., 2014; Iadecola, 2017; Kisler et al., 2017; Sweeney, Ayyadurai & Zlokovic, 2016).

Also, another non-neuronal cell type crucial for inducing HR was recently identified—vascular endothelium. Several kinds of research demonstrated that vascular endothelium could propagate upstream dilations of cerebral vessels (Andresen, Shafi & Bryan, 2006; Chen et al., 2011; Hannah et al., 2011; Iadecola, 2017; Rosenblum, 1986). The in vivo study by Chen et al. (2014), demonstrated that spatially selective endothelial disruption with light-dye treatment in rats somatosensory cortex significantly attenuated the HR by blocking the retrograde dilation. The early stage and the peak of hyperaemia were affected the most, meaning that neurones, astrocytes, pericytes and endothelial cells are all involved in forming HR detected by functional neuroimaging.

There are many other scientific sources regarding cellular, molecular biology and NVC that could be discussed. However, even with the given three *in vivo* examples, it is evident that the main drivers of NVC, and temporal properties of HR associated with it, depends on the spatial location along the cerebral vasculature (Iadecola, 2017). These new findings allow re-evaluating, how spatiotemporal specificity may be improved alongside the technological progress of fNIRS and fMRI. Because ultimately, the non-invasive use of HR is one of the most powerful tools at our disposal to explore human cognition in health and disease.

## Accounting for the Complexity of Cerebrovascular Regulation

The HR describes the empirical observation of a physiological NVC event. It may be detected as an amplitude changes in light absorption (fNIRS) or as a magnetic signal variation (fMRI). In other words, a HR is a spatiotemporal picture of underlying NVC and cerebrovascular regulation at large. To better understand this, some structural and functional properties of cerebrovasculature must be explained.

### Neurovascular unit

**Figure 2:**
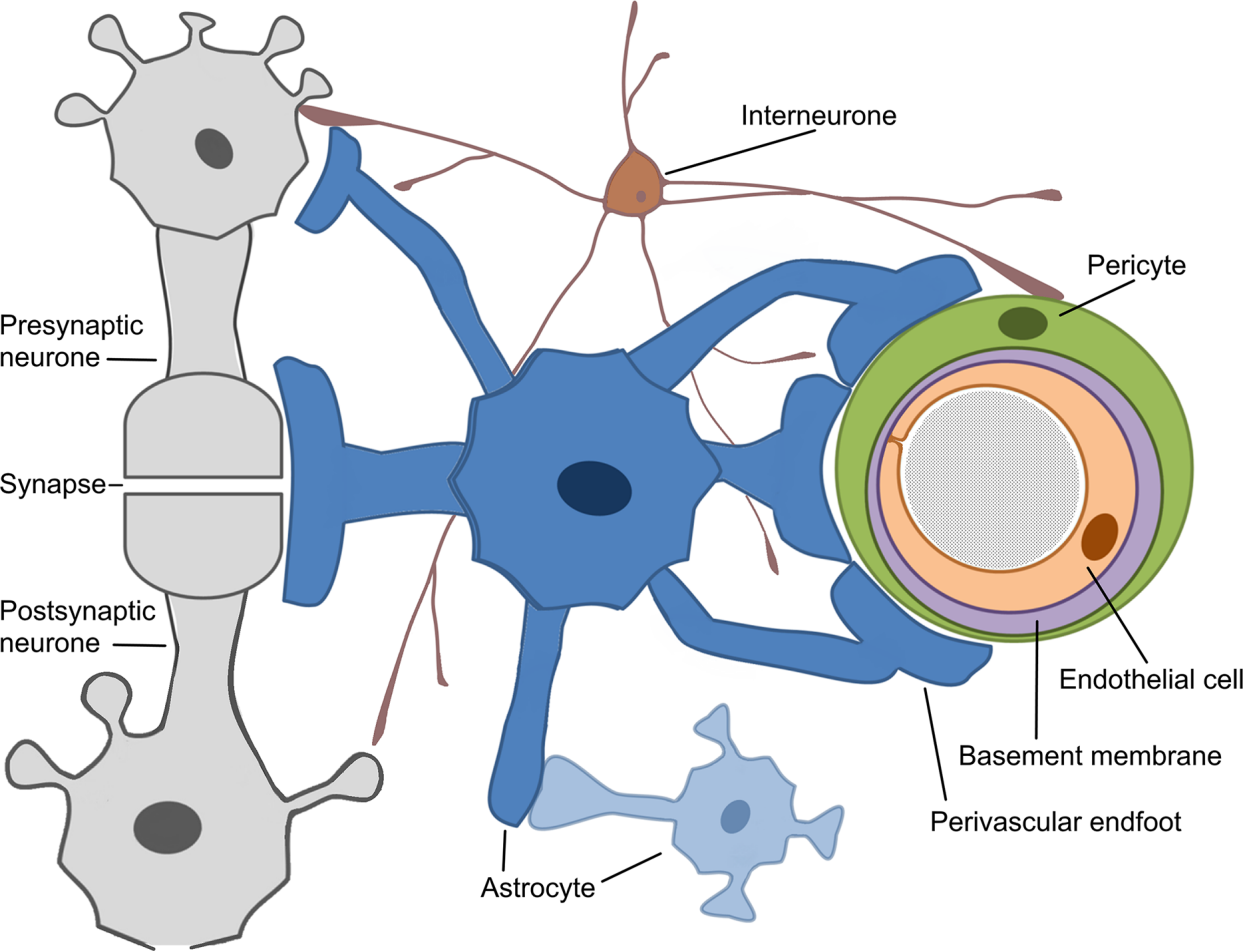
Schematic representation of the Neurovascular Unit (NVU).

The concept of the neurovascular unit (NVU) emerged around 2001 (Iadecola, 2017). The whole mechanism of cerebrovascular regulation can generally be decomposed into several stages (Hamel, 2006; Phillips et al., 2016; Walsh, 2016). The most explored cerebral microcirculation is provided by the structural and functional derivative called NVU, (Attwell et al., 2010; Leybaert, 2005). The neurovascular unit represents the interface between the vascular and neural compartments in the brain and is composed of vascular, glial and neuronal cells such as neurones, astrocytes, endothelial cells and pericytes (Hawkins & Davis, 2005; Sweeney, Ayyadurai & Zlokovic, 2016; Sweeney, Walker-Samuel & Shipley, 2018), (Fig. 2). The NVU is an essential structure for several main processes: formation of neurometabolic coupling (NMC), NVC and formation of the BBB, (Leybaert, 2005; Leybaert et al., 2007; Petzold & Murthy, 2011). NVU may vary in structure and function depending on its location in the brain (Iadecola, 2017; Kowiański et al., 2013; Petzold & Murthy, 2011), thus emphasising the complexity of numerous processes that are involved in maintaining adequate blood flow in the healthy human brain. Neuronal activity in the brain causes two cerebrovascular regulation processes: NMC, which undergoes a substrate exchange between a neurone and an astrocyte and later initiates (but not necessarily) NVC. NVU also supports the BBB coupling but is believed to be unbundled from the already mentioned NBC and NVC, as it regulates the integrity and functions of the BBB (Leybaert, 2005; Sweeney, Ayyadurai & Zlokovic, 2016). New pieces of evidence suggest that another critical component of the NVU are the interneurones that transduce signals from perivascular nerves (Hamel, 2006; Walsh, 2016), (Fig. 2). The crucial role of the perivascular nerves is to regulate the cerebrovascular tone influencing the overall brain perfusion. NVC is then determined by the chemical signals released from the activated perivascular nerves and astrocytes, and together alter the vascular tone to adjust local perfusion in accordance with the brain activity (Hamel, 2006; Walsh, 2016).

### Signal transduction path in the neurovascular unit

With progress in molecular and cellular biology, a conceptual shift in our understanding of cerebral blood flow occurred where astrocytes, previously believed to be passive supporting cells, had been shown to actively participate in many other physiological processes, as well as creating equivalent to neural to the astroglial network, and directly modulating neural activity (Giaume et al., 2010; Kowiański et al., 2013; Scemes & Spray, 2003). For a while, the idea that elevations of calcium concentration in the astrocytes may release transmitters that regulate neuronal and vascular functions was controversial (Barres, 2008; Bezzi et al., 2004; Fiacco, Agulhon & McCarthy, 2009). This changed when numerous contradictions were reported between different studies and had been resolved (Bazargani & Attwell, 2016). Shortly after the discovery that glutamate triggers an increase of intracellular calcium concentration ([Ca^2+^]_*i*_), it was suggested that there might be a mechanism by which calcium signalling propagates towards astrocyte’s endfeet and stimulates the release of vasoactive messengers (Attwell et al., 2010). Vasoactive messengers can cause vasodilatation (most of the neurotransmitters; nitric oxide; prostaglandins; epoxyeicosatrienoic acids; lactate; adenosine etc.) or vasoconstriction (norepinephrine; 20-Hydroxyeicosatetraenoic acid etc.) of arterioles (Giaume et al., 2010; Kowiański et al., 2013; Leybaert, 2005; Petzold & Murthy, 2011; Scemes & Spray, 2003). Current understanding suggests that astrocytes, as well as neurones, should be divided into three spatial compartments, such as processes, soma and endfeet (Bazargani & Attwell, 2016). In this way, the release of specific vasoactive messengers in the endfeet is explained by an overall summation of ([Ca^2+^]_*i*_) in the soma and processes. The response may differ in terms of frequency, kinetics, spatial spreads and interaction with other cellular messengers (Bazargani & Attwell, 2016). As was previously discussed, astrocytes are not the only cells that are involved in HR, but they continue to be considered the main drivers of NVC.

**Figure 3:**
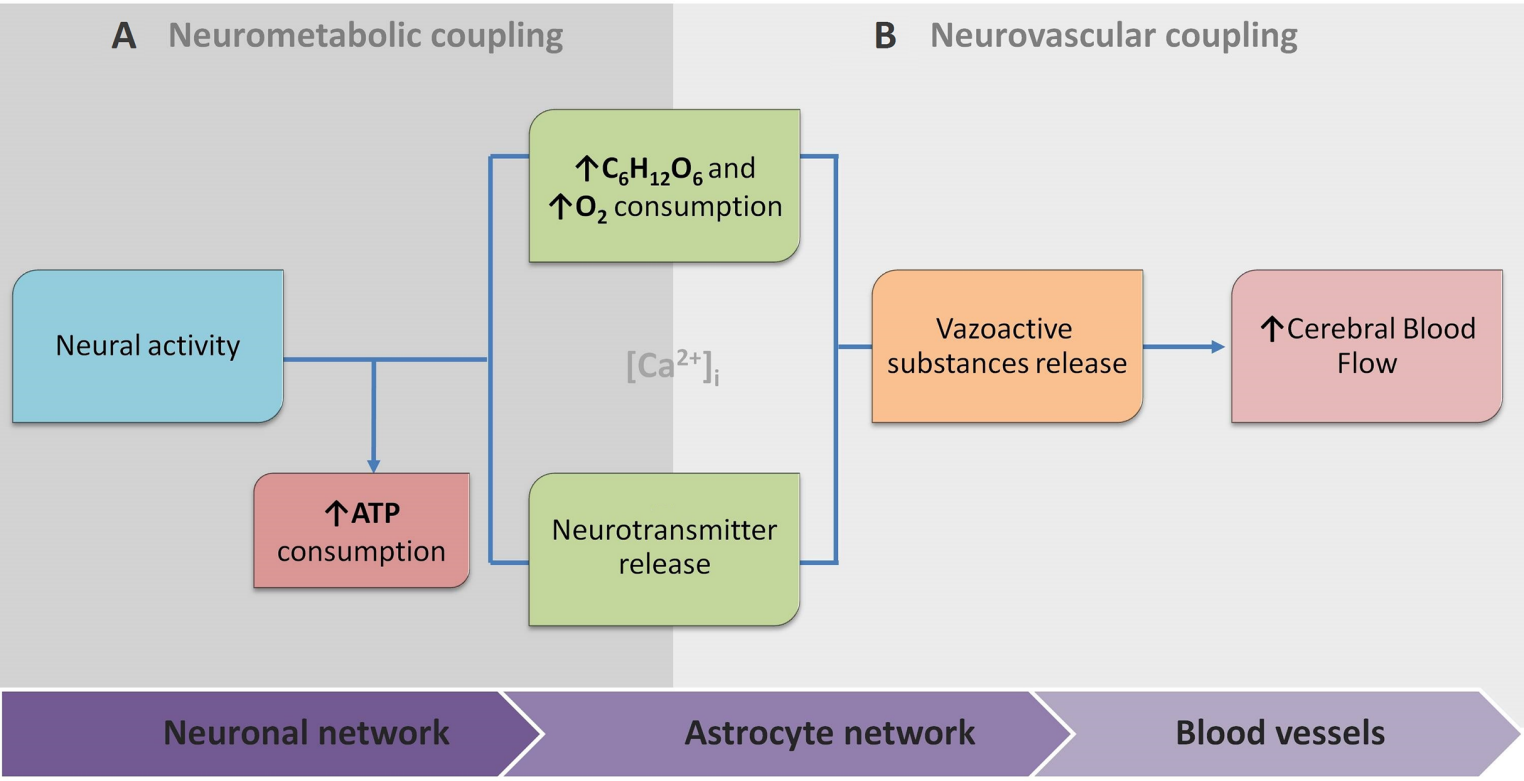
The conceptual biophysical scheme of biological signal transduction path in the Neurovascular Unit (NVU). (A) Neurometabolic coupling (NMC); (B) Neurovascular coupling (NVC). Both neurones (neurotransmitter release) and astrocytes (glucose and oxygen consumption) respond to increased extracellular glutamate, and intracellular calcium to transmit direct and indirect vasoactive signals for the appropriate blood delivery and distribution in the electrically active brain area.

Because of this, the article proposes the conceptual biophysical scheme of the biological signal transduction path in the neurovascular unit (Fig. 3), as a brief adaptation of the classical approach of NVU. Figure 3 summarises how metabolic and physiological events (NMC and NVC accordingly) via calcium concentration elevation cause the HR. This, in turn, can be observed with functional neuroimaging. Note that the conceptual biophysical scheme inevitably assumes neural activity-derived NVC. Nonetheless, recent findings show that astroglial metabolic networks may sustain or suppress neuronal activity (Giaume et al., 2010). For simplicity, this article does not account for it or discuss it thoroughly, as more studies have to be done to generalise new findings (Giaume et al., 2010; Walsh, 2016). However, the idea should be kept in mind for the critical evaluation of current functional neuroimaging methods, and the proposed scheme should be used as a tool for a brief explanation of how signal transduction for cerebrovascular regulation occurs in NVU. Previously mentioned evidence of spatiotemporal specificity of NVU are not included to avoid unnecessary complexity as information about additional players in coupling is still under investigation.

The same complexity of cerebrovascular regulation raised the developmental of numerous approaches, ranging from purely statistical signal processing to biophysical modelling at various levels of detail. Regional hemodynamic changes measured by fNIRS and fMRI are modelled separately because of different aspects of HR that are captured, and sources of noise that are involved. Regarding fNIRS, the extended version of an existing computational model of cerebral physiology, ‘BrainSignals’ should be considered as the most prominent (Caldwell et al., 2016). It incorporates components of (a) hemodynamic; (b) mitochondrial brain metabolism (c) brain oxygen consumption; and (d) scalp hemodynamic. This model also joins hemoglobin-based and the cytochrome c oxidase redox state based measurements (which are out of scope of this article but are promising branch of optical brain measurements). Moreover, the authors compare their empirical model with real measurements, which give promising results in detecting non-linear confounding effects, which are also extensively highlighted by other authors (Tachtsidis & Scholkmann, 2016).

Meanwhile, most models of BOLD response are based solely on cerebral blood flow, cerebral blood volume, and the local metabolic rate of oxygen consumption (Kim & Ogawa, 2012; Toga, 2015). These models depict the transient hemodynamic, and oxygenation changes in the activated cerebral areas also mimic some of the physiological mechanisms of functional hyperemia and are extensively discussed by Huneau, Benali & Chabriat (2015). Authors shortly state, that despite the accumulation of new findings, NVC has surprisingly been forsaken in modelling functional neuroimaging, especially in humans. On the other hand, the field of mathematical modelling of BOLD reached some significant consensus across variables that should be involved in a generative hemodynamic model (using dynamic causal modelling approach), (Havlicek et al., 2015). It involves several different models, such as (a) neuronal; (b) NVC; (c) hemodynamic; and (d) BOLD in a joint model. The approach reflects experimental observations of underlying physiological processes and corresponds well with multimodal experimental datasets (Havlicek et al., 2017).

Meanwhile, technological improvement of neuroimaging techniques allows creating new and more specific models for investigating NVC. For example, a novel *in vivo* study combining imaging of cortical microvascular and mural cell architecture together with mathematical modelling of blood flow and oxygen transport provided new insights on seemingly paradoxical observations in the literature around reduced blood velocity in response to arteriolar constrictions, and found that it might be caused by propagation of constrictions to upstream penetrating arterioles (Sweeney, Walker-Samuel & Shipley, 2018). A similar investigation of cerebral blood flow (CBF) regulation would be inaccessible in a conventional experimental context. In this study, results were achieved by using in vivo collected information for in silico experimentation.

## Why Focused Interdisciplinary Integration Should Matter

How does one determine whether HR under neurological or psychiatric conditions reflects underlying neural activity rather than altered NVC? Does it mean that in many cases additional validation studies linking neuronal activity with NVC might be needed to rely on cognitive inferences derived from functional neuroimaging entirely? A growing body of evidence from animal studies suggests it (Lindauer et al., 2010; O’Herron et al., 2016). Other questions like (i) how new findings of non-neural cell interactions change the interpretation of neural activity derived from previous functional neuroimaging, and (ii) how to distinguish between neural-activity-derived and only glia-activity-derived hemodynamic events, remain open.

**Figure 4:**
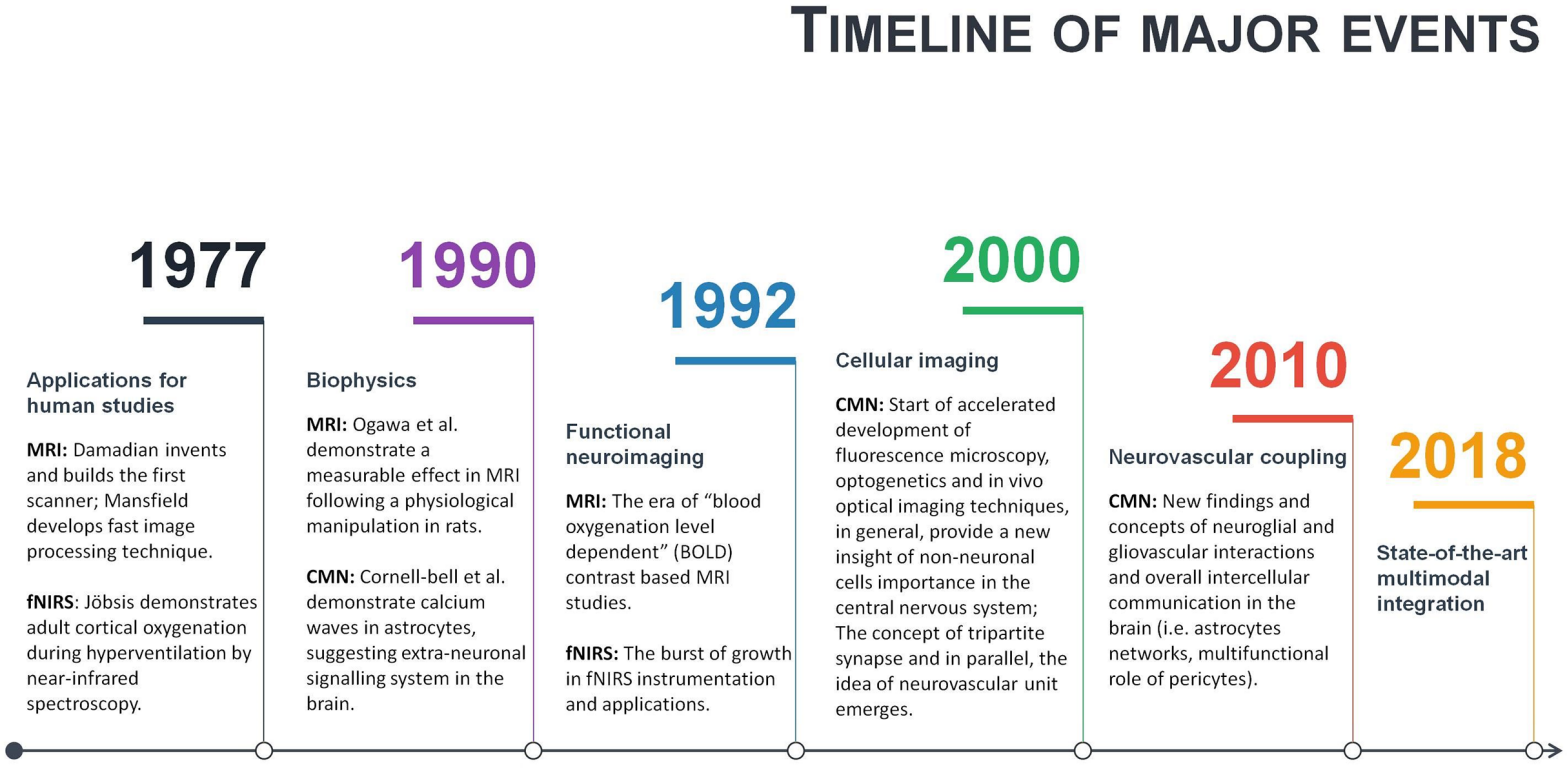
A timeline of magnetic resonance imaging (MRI), functional near-infrared spectroscopy (fNIRS), and cellular and molecular neuroscience (CMN) milestones.

Neuroscience is a multidisciplinary branch of biology, and its scope has broadened over time to include a lot of new and different approaches in many aspects of the nervous system. As a result, neuroscience exploded in a number of interdisciplinary fields such as neurophysiology, cognitive and behavioural neuroscience, computational neuroscience and translational neuroscience research. Somehow, common researchers’ knowledge assumes that in such a broad community of neuroscientists and clinicians, there must be enough professionals dedicating their time and effort to some issues and that necessary integration will occur naturally at the particular threshold. However, some main conceptual challenges may remain daunting due to an unfocused, one-side-driven interdisciplinary integration. Illustrating it with terms of symbiosis: when relationships and interactions between different branches of neuroscience are based more on commensalism (when part A benefits from part B, but B remain unaffected), rather than mutualism (when part A benefits from part B, and *vice versa*). Of course, interdisciplinary integrations are way more complicated, but some relevant patterns may be observed. For example, a few historical moments of fMRI, fNIRS and CMN are given in a single timeline (Fig. 4). As can be seen from Fig. 4, some significant milestones, such as a burst of functional neuroimaging studies using fMRI and fNIRS were achieved simultaneously around 1992. While others, conceptually very important, such as the concepts of the tripartite synapse and neurovascular unit, emerged only around 2000. There is no surprise in the different timing between different neuroscience branch achievements in general. However, even after more than a decade following seminal research in 2000, the NVC is still surprisingly overlooked in functional neuroimaging modelling, especially in humans. This means that current functional neuroimaging inference guidelines are poorly addressing even already known findings of underlying physiological mechanisms of NVC. This renders further interpretations of the HR based functional neuroimaging results ambiguous and entirely reliable only with support from anatomical and electrophysiological studies. As is often the case, the primary concern is not the validity of previous and current studies using functional neuroimaging, but advancements and innovation in the existing paradigm. Ultimately, the non-invasive use of HR is one of the most powerful tools at our disposal to explore human cognition in health and disease, and thus the focused interdisciplinary attention on its accuracy should matter to anyone working or with interests in the field of human brain research.

## Conclusions and Future Perspectives

The significant part of scientific and clinical research production is based on complex mathematical manipulations of neuroimaging data derived from NVC. To improve the overall quality of this production, a complete interpretation of HR should become a number one concern in the field, as it is the primary information source of underlying human brain neurophysiology. However, the amount of available information is growing exponentially, and due to this information overload, researchers’ attention span may be naturally decreased, thus making it a definite obstacle. On the other hand, as was illustrated with the PubMed database search, even within closely related techniques such as fMRI, and fNIRS, there is evident lack of close integration. Moreover, significant results from even more distinct branches of neuroscience such as cellular biology and *in vivo* brain physiology are instead suggested for consideration than provided for implementing in existing CM of human brain function.

One way to overcome it is to stress the concern and make it easy to perceive for broader scientific communities. In accordance, this article fills the gap of a critical view on HR translation into scientific findings and expresses the need for more similarly focused interdisciplinary reviews, as numerous aspects cannot be thoroughly generalised at once. Also, it addresses the need to integrate neurophysiology and computational neuroscience fields to stimulate innovations in neuroimaging by improving an accurate translation of physiological brain signals. At this point, another possible suggestion would be to implement existing machine learning (ML) algorithms for data mining. It would allow meticulous comparison of existing data in CMN studies of NVC, and functional neuroimaging.

In contrast to existing approaches (which use sophisticated algorithms to perform large-scale medical data analysis to search for patterns and predictions in certain brain diseases), attention could be focused on the problem of translating complex physiological phenomena (NVC) to functional neuroimaging (brain maps of HR). This analytical approach in biomedicine is successfully implemented elsewhere (Cao et al., 2018; Ching et al., 2018). In fact, according to latest report of artificial intelligence (AI) use (Shoham et al., 2018), the significant portion of AI-technology-based papers in the USA and Europe tend to be focused on the humanities, and medical and health sciences. Unfortunately, no such attempt was found in the current literature concerning the HR. The existing approaches, from previous (Banaji et al., 2008; Buxton, Wong & Frank, 1998; Caldwell et al., 2016; Friston et al., 2000; Huneau, Benali & Chabriat, 2015; Sotero & Trujillo-Barreto, 2007, 2008), to more recent (Havlicek et al., 2017; Sweeney, Walker-Samuel & Shipley, 2018) models are exploratory, meaning that they try to determine, whether what is being observed might be explained by a currently existing theory. Further, an analytical approach with ML algorithms could be used for patterning and prediction in a conceptually different manner. Despite the notable advantages, it is important to note that applying it would be inevitably challenging; mostly because of data properties, as several different approaches might be needed at once (Cao et al., 2018; Ching et al., 2018). Meanwhile, the author suggests a few general points to discuss on how necessary integration could be initiated:

- Systematic reviews and meta-analyses of previous research studies could be performed selectively on different aspects of NVC and HR (i.e. modality used to investigate, species of a subject, spatial location of interest in the brain volume, goals of research and employed pharmacological agents). The literature search could be expanded with AI algorithms dedicated for the search of relevant scientific content with extensive vocabulary from different neuroscience fields to avoid losing information, when publications conceptually are about the same physiological phenomenon, but due to historical context, or other reasons, is described differently (like terms HR and HR function). This would ensure unbiased (by the investigators’ prior knowledge) collection of relevant publications. As an existing equivalent could be considered AI2 system by the Allen Institute, called ‘Semantic Scholar’, dedicated to finding peer-reviewed research from only trusted, verified sources (https://www.semanticscholar.org). Another example—Elsevier Fingerprint Engine,—the same systems that were applied to explore previously mentioned AI tendency to focus on healthcare. It used a primary set of about 800 keywords relevant to AI (Shoham et al., 2018). Other engines that are not mentioned in this publication may also be used directly or as a prototype. After the initial search on particular aspects of NCV and HR (mostly to make easier the quality check before further analysis), the data could be combined and sliced by any relevant dimension. With a sufficient quantity of information, several different data mining approaches could be possibly applied.
- The initial collection for systematic reviews and meta-analyses could be reused to build a database (or a platform with some user interface) and a unified template for a new data entry could be created, as a suggestion what metadata file that could be associated with a new publication. It would make it easier to import new data and this in turn would increase publication’s impact and visibility. Some concepts of similar systems could also be borrowed and implemented from already existing projects such as the Human Brain Project (https://www.humanbrainproject.eu), or maybe even branch out as a separate compartment of an already existing platform.
- This newly created database could also serve as an information source for any level of computational insight: CM, deep learning, ML, or AI. The database could provide some guidelines for the researchers when searching, or preparing training data for their *in silico* experimentation. The purpose of mentioned algorithms may vary from an automated classification of inputs from living cell microarrays (Jonczyk et al., 2016) to sophisticated machine learning algorithms searching for discrepancies across multimodal studies as in previously given examples.

To sum up, most of the perspective tools are already available and needs only to be implemented with a particular problem. A broader and multi-disciplinary appreciation of NVC could further boost basic and clinical neuroscience. Thus, a reason why it is still not in the frontlines of functional neuroimaging remains debatable. On another hand, this gap in neuroscience requires state-of-the-art scientific research. Because of this, the author invites scientific researches to respond in comments or with a follow-up publication and propose tools or strategies that could be implemented towards accurate translation of the HR.

## Additional Information and Declarations

### Data Availability

The following information was supplied regarding data availability: The research in this article did not generate any data or code (review article).
